# Impact of sleep-related hypoventilation in patients with pleuroparenchymal fibroelastosis

**DOI:** 10.1186/s12931-022-02224-1

**Published:** 2022-10-31

**Authors:** Yuki Yabuuchi, Takefumi Saito, Hitomi Hirano, Mizu Nonaka, Naoki Arai, Kentaro Hyodo, Jun Kanazawa, Yukiko Miura, Shingo Usui, Katsumi Tamura, Tomotaka Kasamatsu, Shuji Oh-ishi, Kenji Hayashihara, Masashi Matsuyama, Nobuyuki Hizawa

**Affiliations:** 1 Department of Respiratory Medicine, National Hospital Organization, Ibaraki Higashi National Hospital, 825 Terunuma. Tokai-Mura, Naka-Gun, Ibaraki, 319-1113 Japan; 2 Department of Clinical Research, National Hospital Organization, Ibaraki Higashi National Hospital, Ibaraki, Japan; 3 Department of Radiology, National Hospital Organization, Ibaraki Higashi National Hospital, Ibaraki, Japan; 4 Department of Radiology, National Defense Medical College, Saitama, Japan; 5 Department of Pulmonary Medicine, Faculty of Medicine, University of Tsukuba, Ibaraki, Japan

**Keywords:** Partial pressure of carbon dioxide, Transcutaneous carbon dioxide, Non-invasive positive pressure ventilation, Chronic pulmonary aspergillosis

## Abstract

**Background:**

Pleuroparenchymal fibroelastosis (PPFE) is a rare fibrosing lung disease with a predilection for the upper lobe and its progression causes hypoventilation, resulting in hypercapnia. Even though the association between sleep-related hypoventilation (SRH) and chronic obstructive pulmonary disease was well documented, its impact in patients with PPFE was not evaluated. The aim of this study is to clarify the impact of SRH on prognosis in PPFE.

**Methods:**

A retrospective review of the medical records of 52 patients with PPFE who underwent transcutaneous carbon dioxide monitoring during sleep was done.

Patients were stratified into SRH (n = 28) and non-SRH (n = 24) groups based on American Academy of Sleep Medicine criteria. The impact of SRH on the prognosis of PPFE, as well as the clinical factors and comorbidities of PPFE associated with SRH, were evaluated.

**Results:**

Forced expiratory volume in the first second (FEV_1_), forced vital capacity (FVC), total lung capacity (TLC), and carbon monoxide diffusing capacity (DLco) in the SRH group were significantly lower than the non-SRH group (P < .01). Chronic pulmonary aspergillosis (CPA) was found at a higher rate in the SRH group (P = .02). The median survival time for SRH patients was 330 days, whereas roughly 80% of non-SRH patients were alive during the 3-year observation period (P < .01). Body mass index was a significant prognostic factor in PPFE patients with SRH (HR .78; 95% CI; .64–.94; P < .01). Home oxygen therapy (HOT) during the day and noninvasive positive pressure ventilation (NPPV) at night while sleeping tended to improve prognosis in the SRH group, as indicated by HR of .25 (P = .07).

**Conclusions:**

SRH may be a poor prognostic factor for PPFE. Additionally, SRH may modify susceptibility to Aspergillosis in patients with PPFE. HOT plus NPPV may improve the disease outcomes in patients with SRH.

## Background

Pleuroparenchymal fibroelastosis (PPFE) is characterized by the progression of fibrotic changes predominantly in the bilateral upper lobes and is defined as a rare interstitial lung disease in idiopathic interstitial pneumonia (IIP) [1, 2]. PPFE sometimes demonstrates type 2 respiratory failure from an early stage, in the form of increase in the partial pressure of carbon dioxide (PaCO_2_), which is distinctly different from other IIPs [2, 3]. The mechanism of these changes has not been elucidated, but may be related to fibrotic changes in the bilateral upper lobes and limited mobility of the chest wall [2].

Sleep-related hypoventilation (SRH) was one of the sleep-related respiratory disorders described in the International Classification of Sleep Disorder (ICSD) 3^rd^ edition as the impairment of ventilation during sleep, resulting in the elevation of the PaCO_2_ [4, 5]. The American Academy of Sleep Medicine (AASM) suggested the following criteria for SRH: PaCO_2_ during sleep increases > 55 mmHg for ≥ 10 min or PaCO_2_ during sleep increases > 10 mmHg compared with awake supine values exceeding 50 mmHg for ≥ 10 min [4]. SRH is subdivided into subtypes and SRH due to a medical disorder [5] is subsequently induced from chronic respiratory diseases presenting with thoracic deformity and respiratory disease consisting of the lung parenchyma, airways, or pulmonary vascular disorders [5, 6], which likely occur in PPFE. Some studies have investigated the association between SRH and chronic obstructive pulmonary disease (COPD) [6–8]. However, as far as we know, studies evaluating the impact of SRH on PPFE are scarce in the published literature. In the current study, we evaluated the effect of secondary SRH on survival outcomes in patients with PPFE.

## Methods

### Study population

We retrospectively reviewed the medical records of 52 consecutive patients diagnosed with PPFE at a tertiary care center who were assessed with transcutaneous carbon dioxide monitoring (PtCO_2_) during sleep over a span of three years. The study protocol was approved by the Institutional Ethics Committee (No. 2019-027). All procedures in this study were performed according to the ethical standards of the Institutional Research Committee and adhered to the tenets of the Helsinki Declaration (1964). The opt-out method was used for patient consent instead of obtaining informed consent.

### Measurement

Parameters such as age, sex, body mass index (BMI), smoking history, serum levels of Krebs von den lungen-6 (KL-6), arterial blood gas analysis (ABG), and respiratory comorbidities were evaluated. The pulmonary function test (PFT) (CHESTAC-8900; Chest, Tokyo, Japan) and diffusing capacity for carbon monoxide (DL_CO_) using the single-breath method (CHESTAC-8900) were also measured. The PFT was conducted according to the American Thoracic Society (ATS) recommendations for acceptability and reproducibility [9]. Using spirometry, forced expiratory volume in one second (FEV_1_), forced vital capacity (FVC), FEV_1_/FVC, VC, total lung capacity (TLC), residual volume (RV), DL_CO,_ and DL_CO_/ alveolar volume (V_A_) were evaluated. The values of FEV_1_, FVC, VC, TLC, RV, RV/TLC, DL_CO,_ and DL_CO_/V_A_ were expressed as percentages of the predicted values. Additionally, the gender-age-physiology (GAP) score was evaluated, as the prognostic value for idiopathic pulmonary fibrosis (IPF) [10].

ABG samples were collected when a patient was awake, in a resting state, and in the supine position. The levels of PaCO_2_ and hydrogen carbonate (HCO_3_^−^) were evaluated within the normal pH range of 7.35 to 7.45. To analyze nocturnal PtCO_2_ during sleep, a transcutaneous blood gas monitoring system (TOSCA®) was used. The sensor worn on the earlobe had a built-in heating system that raised the skin temperature to 42 °C to increase both blood flow and CO_2_ diffusion and detects CO_2_ value [11]. Generally, skin CO_2_ is higher than blood PaCO_2_; therefore, PtCO_2_ was carbureted, thereby calculating approximate PaCO_2_ [11]. Modern PtCO_2_ monitoring systems are extremely precise and have an accuracy level within that of PtCO_2_ and PaCO_2_ [8, 11, 12]. PaO_2_ and the mean and maximum values of PtCO_2_ during sleep were evaluated.

To analyze the association between SRH and platythorax, a flat chest index was estimated [13]. For each patient, clinical events and survival were followed for 3 years after the presence of SRH was evaluated. Clinical events included the occurrence of respiratory adverse events, cause of death, introduction of drug therapies (nintedanib, pirfenidone, prednisolone, and immunosuppressant), non-invasive positive pressure ventilation (NPPV) during sleep, and home oxygen therapy (HOT) during the day. A medical team consisting of pulmonologists (YY, TS) and radiologists (TK, KT) with over 20 years of experience evaluated the high-resolution computed tomography (HRCT) scans. The clinical course of the patient was reviewed by the attending physician, a respiratory specialist. Since no significant differences in the clinical course or outcome have been reported between idiopathic and secondary PPFE [14], this study does not include a detailed examination of the etiology of individual cases.

### GAP score

The GAP score was classified 0–3 for Stage I, 4 and 5 for Stage II, and 6–8 for Stage III, and as the stage progressed, the prognosis became worse in IPF [10]. Based on recent literature, the GAP score can also be applied in PPFE [15, 16]; therefore, wherever the data were available, participants were stratified as Stage I – III.

### Flat chest index

It has been reported that the thoracic cage becomes flat with the progression of PPFE, resulting from fibrotic collapse in the upper lobes [13, 17]. These changes may induce compensatory overinflation in the lower lobes, which reflects an increase in the RV/TLC [2, 17]. The flat chest index presented the degree of platythorax, defined as the ratio of the anteroposterior diameter to the transverse diameter in the thoracic cage at the level of the sixth thoracic vertebra on chest CT [13]. In this study, this parameter was measured in the participants who underwent chest CT.

### Diagnosis of PPFE

PPFE was mainly diagnosed by HRCT based on Reddy’s criteria [18] and clinical findings, as mentioned in previous studies [14, 15, 19, 20]. Pathological findings, wherever available were used as references; however, lung biopsy may carry a high risk of secondary pneumothorax and surgical lung biopsy is not recommended [19, 20]. Enomoto et al. [20] defined the clinical diagnostic criteria of idiopathic PPFE as follows: (1) Radiological findings of definite PPFE (bilateral subpleural dense consolidation with or without pleural thickening and/or a reduction of volume in the upper lobes). (2) Radiological disease progression (an increase in upper lobe consolidation with or without pleural thickening and/or reduction of volume in the upper lobes). (3) Exclusion of differential lung diseases (such as connective tissue disease-related interstitial lung disease, chronic hypersensitivity pneumonitis, pulmonary sarcoidosis, pneumoconiosis, and active pulmonary infection). We did not distinguish between idiopathic and secondary PPFE in this study; therefore, the third criterion was not used.

Comorbidities assessed in the study included COPD, bronchial asthma (BA), radiological lower-lobe interstitial lung disease (ILD) in usual interstitial pneumonia (UIP), nonspecific interstitial pneumonia (NSIP), and chronic pulmonary aspergillosis (CPA). The diagnosis of COPD, BA, and CPA was based on established criteria [21–23]. UIP and NSIP on HRCT were also based on the established criteria [24].

### Diagnosis of SRH

SRH was diagnosed using AASM criteria [4]. Some reports have shown a strong correlation between PtCO_2_ and PaCO_2_ [11, 12]; therefore, levels of PaCO_2_ during sleep were substituted by PtCO_2_, while awakening PaCO_2_ was measured by arterial blood gas (ABG) in a resting state and in the supine position. In this regard, the SRH criteria in the AASM were modified to PtCO_2_ increasing > 55 mmHg for ≥ 10 min or PtCO_2_ increasing > 10 mmHg compared with awake supine values in PaCO_2_ of ABG exceeding 50 mmHg for ≥ 10 min. PtCO_2_ was performed for PPFE patients who had been suspected of having ventilation disturbance based on symptoms, results of ABG, pulmonary function tests (PFT), chest X-ray (CXR), and HRCT.

To assess the condition of each patient over the course of the disease, we excluded patients with acute respiratory failure as follows: (1) patients were in the intensive care unit (ICU), (2) acute changes in the condition of the patient in the time period from examining PtCO_2_ to ABG, (3) acute respiratory diseases that were complicated during or just before PtCO_2_, (4) tranquilizers were used before PtCO_2_, and (5) patients who were managed with mechanical ventilation by intubation, non-invasive positive pressure ventilation (NPPV), continuous positive airway pressure, or nasal high flow oxygen therapy.

### Data analysis

All data were presented as medians (interquartile ranges) for continuous variables and as numbers or percentages for categorical variables. The comparison between two categorical variables was evaluated using the chi-squared or Fisher’s exact test. The Mann–Whitney U-test was used to compare continuous variables. For analyzing overall survival for 3 years after TOSCA® evaluation, the Kaplan–Meier method was used to present the estimated survival probabilities and the comparison of survival rates was made by log-rank test. Additionally, univariate and multivariate Cox proportional hazards models were applied to analyze independent predictors of survival. Stepwise regression was used to select explanatory variables in the multivariate analysis. The predictive factors were age, smoking history, BMI, male gender, PaCO_2_, HCO_3_^−^, PtCO_2_ mean, PtCO_2_ max, %FEV_1_, FEV_1_/FVC, %VC, %FVC, %TLC, %RV, %RV/TLC, %DL_CO_, %DL_CO_/V_A_, KL-6, GAP score stage, flat chest index, and HOT + NPPV.

In this study, all variables with P < .05 were defined as statistically significant. Data analysis was performed using R software (version 4.0.3; R Foundation for Statistical Computing, Vienna, Austria).

## Results

Among 52 consecutive patients with PPFE, 28 (53.8%) were diagnosed as having SRH. The clinical characteristics of the study participants are listed in Table 1. The mean age, sex, smoking history, body mass index, and KL-6 of the enrolled patients had no differences between the SRH and non-SRH groups. As a matter of course, both mean and max PtCO_2_ in the SRH group were higher than in the non-SRH group with a significant statistical difference. Differences were also observed in the results of pulmonary function tests and DL_CO_ using the single-breath method. The SRH group progressed with pulmonary restrictive changes, namely decreased VC, and FVC, compared with the non-SRH group. The FRC, TLC, and RV in the SRH group were lower than those in the non-SRH group, indicating that the lung volume in the SRH group tended to shrink significantly compared to the non-SRH group. Moreover, the pulmonary diffusion capacity, DL_CO,_ and DL_CO_/V_A_, of the SRH group were also worse than that of the non-SRH group, which likely highlighted the destruction of the alveolar wall and pulmonary capillary bed due to disease progression. Additionally, the results of ABG also reflected the difference between the SRH and the non-SRH group; levels of PaCO_2_ and HCO_3_^−^ within the normal range of pH in the SRH group were obviously higher than those of the non-SRH group, which reflected chronic alveolar hypoventilation.

**Table 1:** Clinical charactaristics of SRH (n = 28) and non SRH (n = 24) in PPFE

	Total (n = 52)	SRH(n = 28)	non SRH(n = 24)	p-value
Age (year)	76 (12.2)	76 (13.3)	76 (13.2)	.22
Gender (male/female)	33/19	19/9	14/10	.23
Smoking history	23 (44.2%)	12 (42.8%)	11 (45.8%)	1.00
Body mass index (kg/m^2^)	17.5(4.9)	16.7(4.8)	19.1(4.7)	.19
Arterial blood gas during awakening				
PaCO_2_	46 (10.8)	51.5 (13.6)	42.5 (6.6)	< .01
HCO_3_^−^	30 (5.6)	32.8 (5.6)	27.4 (4)	< .01
PtCO_2_ during sleep				
Mean	53 (11)	58.6 (10.6)	47.4 (4.3)	< .01
Max	60 (12.5)	67 (15.5)	54 (3.8)	< .01
Pulmonary function test				
%FEV_1_	50.9 (28.6)	42.9 (18.4)	62.9 (29.1)	< .01
FEV_1_/FVC	86.8 (19.8)	87.6 (20)	85.2 (27.8)	.29
%VC	52.1 (27.8)	37.1 (15.6)	60.7 (20.9)	< .01
%FVC	52.2 (32.6)	36.3 (18.5)	63.4 (18.5)	< .01
%TLC	74.1 (34)	52.7 (27.9)	84.4 (17.9)	< .01
%RV	92.9 (46.2)	83.1 (34.8)	119.1 (60.5)	< .01
%RV/TLC	143.1 (37.6)	142.7 (37.2)	143.6 (38.2)	.58
%DL_CO_	60.7 (47.2)	32.5 (43.2)	66.8 (25.3)	< .01
%DL_CO_/V_A_	74.1(41)	71.9 (43.3)	77.4 (37)	.22
Blood examination				
KL-6	373 (319)	457 (394.2)	303 (331)	.12
GAP score stage (I/II/III)	17/20/9	6/11/8	11/9/0	< .01
Flat chest index	.56 (.1)	.55 (.1)	.6(.1)	.62

The data are presented as n (%) or median (interquartile range)

*SRH* sleep-related hypoventilation, *PPFE* pleuroparenchymal fibroelastosis, *BMI* body mass index, *PaCO*_*2*_ arterial carbon dioxide partial pressure during awakeing, *PtCO*_*2*_ transcutaneous carbon dioxide monitoring during sleep, *HCO*_*3*_^*−*^ hydrogen carbonate in awakening arterial blood gas analysis, *%FEV*_*1*_%forced expiratory volume in one second, *%FVC* %forced vital capacity, *FEV*_*1*_*/FVC* forced expiratory volume in one second/forced vital capacity, *%VC* %vital capacity, *%TLC* %total lung capacity, *%RV* %residual volume, *%DL*_*CO*_ %diffusing capacity for carbon monoxide, *%DL*_*CO*_*/V*_*A*_ %diffusing capacity for carbon monoxide/alveolar volume, *KL-6* Krebs von den lungen-6, *GAP* gender-age-physiology

A comparison of baseline comorbidities between the SRH and non-SRH groups in PPFE patients is shown in Table 2. A higher frequency of CPA was found in the SRH group.

**Table 2:** Comobidities of SRH (n = 28) and non SRH (n = 24) in PPFE

	Total (n = 52)	SRH (n = 28)	non SRH (n = 24)	p-value
COPD	7	4	3	1
BA	3	2	1	1
Lower lobe ILD (UIP/NSIP)	4/7	3/2	1/5	.22
CPA	6	6	0	.02
Pneumothorax	10	6	4	.73
Bacterial pneumonia	9	4	5	.72

The data are presented as n

*SRH* sleep-related hypoventilation, *PPFE* pleuroparenchymal fibroelastosis, *COPD* chronic obstructive pulmonary disease, *BA* bronchial asthma, *ILD* interstitial lung disease, *NSIP* nonspecific interstitial pneumonia, *CPA* chronic pulmonary aspergillosis

Lower lobe ILD demonstrated UIP or NSIP pattern in HRCT

**Fig. 1:**
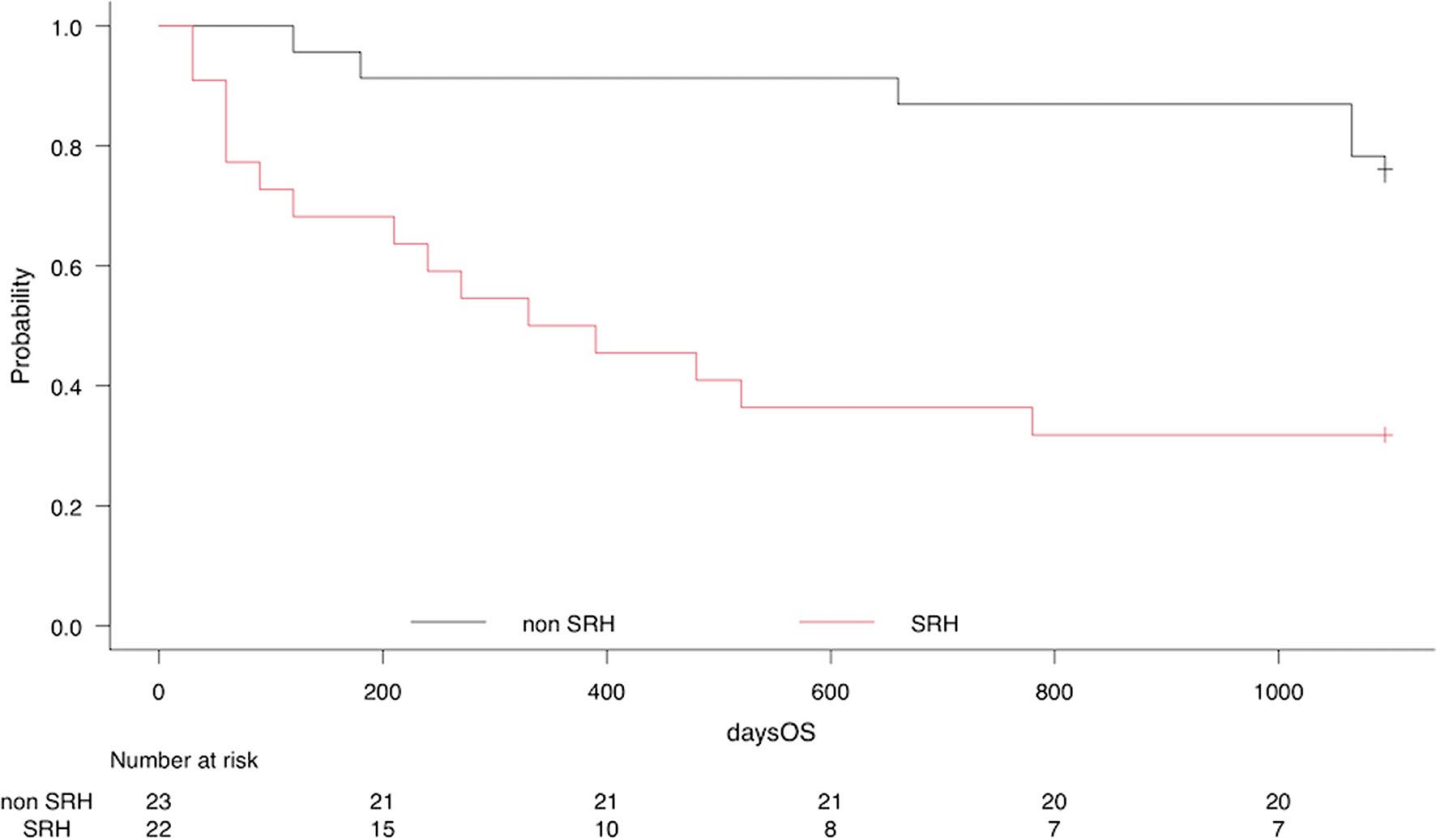
Kaplan–Meier survival curves in PPFE patients with SRH vs those with non SRH. *PPFE* pleuroparenchymal fibroelastosis, *SRH* sleep-related hypoventilation

In patients with SRH in PPFE (n = 28), there were some events including death during the follow-up period (Table 3). Pneumothorax was the most common complication (n = 7), followed by bacterial pneumonia (n = 4) and CPA (n = 3). Sixteen patients died (57.1%), and the cause of death was the worsening of type-2 respiratory failure (68.7%). The causes of death in the remaining patients included pneumothorax (n = 3), acute exacerbation (n = 1), and sepsis (n = 1). Kaplan–Meier survival curves in the SRH and the non-SRH groups are presented in Fig. 1, which showed a significant difference (P < .01).

**Table 3:** Events after diagnosis of SRH in PPFE

Events	n
Respiratory complications	
Pneumothorax	7 (25.0%)
Bacterial pneumonia	4 (14.3%)
CPA	3 (10.7%)
Acute exacerbation	1 (3.5%)
Death at the end of follow up	16 (57.1%)
Reasons of death	
Worsening of hypercapnia	11 (68.7%)
Pneumothorax	3(18.7%)
Acute exacerbation	1 (6.2%)
Sepsis	1 (6.2%)
Introducing drug therapy	
Nintedanib	3 (10.7%)
Alive after introducing	0 (0%)
Death after introducing	3 (100%)
Pirfenidone	2 (7.1%)
Alive after introducing	1 (50%)
Death after introducing	1 (50%)
Prednisolone	0 (0%)
Immunosuppressant (tacrolimus)	1 (3.5%)
Alive after introducing	1 (100%)
Death after introducing	0 (0%)
Introducing home ventilator	
HOT + NPPV	9 (32.1%)
Alive after introducing	3 (33.3%)
Death after introducing	5 (55.5%)
Untraceable because of transferring another hospital	1 (11.1%)

The data are presented as n (%)

*SRH* sleep-related hypoventilation, *PPFE* pleuroparenchymal fibroelastosis, *CPA* chronic pulmonary aspergillosis, *HOT* + *NPPV* introducing home oxygen therapy (HOT) during daytime plus non-invasive positive pressure ventilation (NPPV) during sleep

During the three years follow-up period, there were 16 deaths in 23 patients with SRH and 4 deaths in 22 traceable patients with non-SRH. Seven patients were untraceable because of transfer to another hospital or self-interruption of medical check-ups. The median survival time in the SRH group was 330 days while about 80% patients are alive in the follow-up period in the non-SRH group (log-rank P < .01). The Cox proportional hazard ratio demonstrated a hazard ratio (HR) of 5.36; 95% confidence interval (CI); 1.94–14.82; P < .01, which identified SRH as a prognostic factor in PPFE.

Univariate Coxproportional hazards models were used to identify the prognostic factors in patients with SRH in PPFE, as listed in Table 4. The analysis demonstrated that BMI (HR .77; 95% CI; .64-.94; P < .01) was a significant prognostic factor in patients with SRH in PPFE. In addition, 9 patients were managed with home oxygen therapy (HOT) during the daytime and NPPV during sleep. Five of the 9 patients (55.5%) died within the follow-up period (Table 3), whereas 11 patients (57.9%) died among the 14 who did not receive this therapy (P = .07).

**Table 4:** Univariate analysis of prognostic factors of SRH in PPFE

	HR [95% CI]	p-value
Age	1.02 [.98–1.06]	.37
Smoking history	2.19 [.81–5.92]	.12
BMI	.77 [.64–.94]	< .01
Gender male	1.27 [.43–3.74]	.66
PaCO_2_	1.01 [.96–1.05]	.76
HCO_3_^−^	1.08 [.95–1.24]	.25
PtCO_2_ mean	.98 [.94–1.03]	.45
PtCO_2_ max	.99 [.96–1.03]	.69
%FEV_1_	.96 [.25–3.68]	.95
FEV_1_/FVC	1.01 [.96–1.07]	.66
%VC	1.00 [.97–1.04]	.83
%FVC	.99 [.96–1.03]	.73
%TLC	1.02 [.98–1.06]	.35
%RV	1.01 [.99–1.04]	.31
%RV/TLC	1.00 [.98–1.02]	.77
%DL_CO_	1.00 [.98–1.03]	.88
%DL_CO_/V_A_	.99 [.97–1.02]	.63
KL-6	1.00 [.99–1.01]	.94
GAP score stage	1.07 [.54–2.13]	.85
Flat chest index	.42 [.01–50.00]	.72
HOT + NPPV	.25 [.06–1.12]	.07

The data are presented as hazard ratio [95% confidence interval]

*HR* hazard ratio, *CI* confidence interval, *SRH* sleep-related hypoventilation, *PPFE* pleuroparenchymal fibroelastosis, *BMI* body mass index, *PaCO*_*2*_ arterial carbon dioxide partial pressure in awaking, *PtCO*_*2*_ transcutaneous carbon dioxide monitoring during sleep, *HCO*_*3*_^*−*^ hydrogen carbonate in awaking arterial blood gas analysis, *%FEV*_*1*_%forced expiratory volume in one second, *%FVC* %forced vital capacity, *FEV*_*1*_*/FVC* forced expiratory volume in one second/forced vital capacity, *%VC* %vital capacity; *%TLC* %total lung capacity, *%RV* %residual volume, *%DL*_*CO*_ %diffusing capacity for carbon monoxide, *%DL*_*CO*_*/V*_*A*_ %diffusing capacity for carbon monoxide, *KL-6* Krebs von den lungen-6, *GAP* gender-age-physiology, *HOT* + *NPPV* introducing home oxygen therapy (HOT) in daytime plus non-invasive positive pressure ventilation (NPPV)

Furthermore, univariate and multivariate Cox proportional hazards models were used to construct prognostic models for all patients with PPFE, as shown in Table 5. In univariate analysis, the following factors demonstrated significant differences as prognostic factors, respectively; BMI (HR .74; 95% CI; .65–.85; P < .01), PaCO_2_ (HR 1.05; 95% CI; 1.02–1.09; P < .01), HCO_3_^−^ (HR 1.23; 95% CI; .65–.85; P < .01), PtCO_2_ mean (HR 1.04; 95% CI; 1.01–1.07; P = .02), PtCO_2_ max (HR 1.03; 95% CI; 1.01–1.06; P = .01), %VC (HR .96; 95% CI; .94-.99; P = .01), %FVC (HR .96; 95% CI; .94-.99; P < .01),

**Table 5:** Univariate and multivariate analysis of prognostic factors of total PPFE

	Univariate analysis		Multivariate analysis	
	HR [95% CI]	p-value	HR [95% CI]	p-value
Age	1.00 [.97–1.04]	.84		
Smoking history	1.11 [.47–2.61]	.81		
BMI	.74 [.65–.85]	< .01	.68 [.56–.83]	< .01
Gender male	1.02 [.42–2.46]	.66		
PaCO_2_	1.05 [1.02–1.09]	< .01		
HCO_3_^−^	1.23 [1.09–1.38]	< .01		
PtCO_2_ mean	1.04 [1.01–1.07]	.02		
PtCO_2_ max	1.03 [1.01–1.06]	.01		
%FEV_1_	.98 [.95–1.00]	.08		
FEV_1_/FVC	1.05 [1.00–1.09]	.05		
%VC	.96 [.94–.99]	.01		
%FVC	.96 [.94–.99]	< .01		
%TLC	.98 [.96–1.00]	.05		
%RV	.99 [.99–1.00]	.14		
%RV/TLC	1.00 [.98–1.02]	.92		
%DL_CO_	.98 [.96–.99]	.04		
%DL_CO_/V_A_	.98 [.96–1.00]	.10		
KL-6	1.00 [.99–1.01]	.52		
GAP score stage	1.5 [1.12–2.02]	< .01	2.44 [1.09–5.46]	.03
Flat chest idex	.01 [.01–1.14]	.06		
HOT + NPPV	1.15 [.39–3.43]	.08		
SRH	5.36 [1.94–14.82]	< .01	4.84 [1.31–17.93]	.02

The data are presented as hazard ratio [95% confidence interval]

*HR* hazard ratio, *CI* confidence interval, *SRH* sleep-related hypoventilation, *PPFE* pleuroparenchymal fibroelastosis, *BMI* body mass index, *PaCO*_*2*_ arterial carbon dioxide partial pressure in awaking, *PtCO*_*2*_ transcutaneous carbon dioxide monitoring during sleep, *HCO*_*3*_^*−*^ hydrogen carbonate in awaking arterial blood gas analysis, *%FEV*_*1*_%forced expiratory volume in one second, *%FVC* %forced vital capacity, *FEV*_*1*_*/FVC* forced expiratory volume in one second/forced vital capacity, *%VC* %vital capacity, *%TLC* %total lung capacity, *%RV* %residual volume, *%DL*_*CO*_ %diffusing capacity for carbon monoxide, *%DL*_*CO*_*/V*_*A*_ %diffusing capacity for carbon monoxide, *KL-6* Krebs von den lungen-6, *GAP* gender-age-physiology, *HOT* + *NPPV* introducing home oxygen therapy (HOT) in daytime plus non-invasive positive pressure ventilation (NPPV)

%DL_CO_ (HR .98; 95% CI; .96–.99; P = .04), GAP score stage (HR 1.50; 95% CI; 1.12–2.02; P < .01), and SRH (HR 5.36; 95% CI; 1.94–14.82; P < .01). For these factors, stepwise regression was performed to select the explanatory variables for multivariate Cox proportional hazards models. As a result, GAP score stage, BMI, and SRH were selected. The results of the multivariate analysis (Table 5) revealed significant differences in the three factors as follows:

GAP score stage (HR 2.44; 95% CI; 1.09–5.46; P = .03), BMI (HR .68; 95% CI; .56-.83; P < .01), and SRH (HR 4.84; 95% CI; 1.31–17.93; P = .02).

The results indicated that SRH with BMI and GAP score stage may be useful factors as prognostic models in PPFE.

## Discussion

This study aimed to determine whether the complication of SRH in patients with PPFE has an impact on their survival. To the best of our knowledge, there is no research on the relationship between PPFE and SRH. In this study, the SRH group had poor prognosis than the non-SRH group. Worsening of hypercapnic chronic respiratory failure was common reason of death in PPFE [20, 25]. In addition, a subgroup of advancing disease types, the so-called progressive PPFE phenotype, has been recognized [2, 26]. The feature of progressive PPFE disease is characterized by the presence of co-existing usual interstitial pneumonia and complications of pneumothorax or pneumomediastinum, and the median survival described in this phenotype is less than 5 years [2]. In this study, we identified CPA as the comorbidity, presenting a significant difference between the SRH and non-SRH groups (Table 2). Although several studies have reported that fibrotic lesions of PPFE have susceptibility to Aspergillus infection [26, 27], the causal relationship between SRH and Aspergillus infection requires further investigation.

In general, the progression of the fibrotic process in PPFE tends to worsen hypercapnic respiratory failure, which is a characteristic clinical course compared to other ILDs [3, 25]. The mechanisms of hypoventilation in PPFE have not been established; however, the following characteristic conditions in PPFE may be associated: upper lobe predominantly fibrotic changes of the visceral pleura and subpleural parenchyma and platythorax with slender [1, 2, 13]. PFT in intra- and extra-thoracic deformities demonstrates characteristic changes with worsening of FVC and a mild decrease in TLC and an increase in the ratio of RV/TLC [1, 2, 14, 15]. The diffusion capacity of DL_CO_ also decreases while preserving DL_CO_/V_A_ [1, 2, 25, 26]. In this study, as shown in Table 1, PFT and diffusing capacity demonstrated that FVC, TLC, and DL_CO_ in the SRH group were significantly lower than those in the non-SRH group. These changes strongly affected the GAP score stage, with the SRH group demonstrating an advanced stage with significant differences. In contrast, the ratio of RV/TLC was high and DL_CO_/V_A_ was low in both groups, without significant differences. There was no difference between the SRH and the non-SRH groups in the flat chest index presenting with platythorax, which may have no association with hypercapnia. These results indicate that the presence of SRH reflects more restrictive and shrinking lung states in PPFE. During sleep, these changes may occur even in the early stages of PPFE [1, 5, 25]. Generally, in rapid eye movement (REM) sleep, a state of mild hypoventilation and upper airway stenosis is caused by atonia of the respiratory musculature, but not the diaphragm [5, 6]. In PPFE, the progression of fibrosis and pleurodesis in the upper lobe may restrict the disturbance of diaphragmatic movements [1, 25, 27]. These conditions may impair ventilation, resulting in the development of hypercapnia during REM sleep.

It has been reported that NPPV may improve diurnal and nocturnal PaCO_2_ and probably survival, especially in COPD [5–7]. While NPPV can be adapted to PPFE remains controversial, in this study, NPPV was introduced in nine cases, and three patients were alive during the follow-up period. In univariate Cox hazard proportional models, although no significant difference was proven, HOT in daytime plus NPPV in sleep may improve the prognosis of PPFE patients with SRH, as presented in HR of .25. Given that NPPV plus oxygen supplementation during sleep for hypercapnia in COPD improved quality of life and daytime PaCO_2_ compared with oxygen supplementation alone [28], this approach may also aid in PPFE. The optimal setting of NPPV for PPFE patients with SRH remains uncertain, however. Furthermore, the risk of pneumothorax in NPPV has been reported [29, 30]; therefore, deliberate discussions will be required before NPPV is introduced.

### Limitations

The present study had several limitations. First, this study was performed at a single center with a relatively small sample size and had a retrospective study design. Future multicenter prospective studies with adequate sample sizes will be required to validate the data obtained. Second, there may be a small gap between PaCO_2_ and PtCO_2_ measured using the TOSCA® during sleep. In a single-center, 46-comparison study, Kelly et al. found that the average difference between PaCO_2_ and PtCO_2_ was 6.1 mmHg [31]. PtCO_2_ has been shown to have a slight positive bias compared to PaCO_2_ [7]. In the future, monitoring systems for PtCO_2_ during sleep need to be further improved in terms of accuracy. Third, it has not been revealed whether CPA was one of the causes of secondary PPFE, however, there were some reports indicating that CPA associated with construction of PPFE [2, 27]. In this study, six patients had CPA when they were diagnosed as SRH. Those patients fulfilled PPFE criteria, therefore, they were included in 28 PPFE patients with SRH. Fourth, since electroencephalography was not performed concurrently with TOSCA® during sleep, sleep stages were not evaluated. The analysis of the effect of REM sleep on SRH in patients with PPFE may be important; therefore, the addition of electroencephalography is recommended in future studies.

## Conclusion

This study indicates that SRH is a poor prognostic factor for PPFE. Progression of pulmonary fibrosis and reduction in lung volume may be associated with SRH in patients with PPFE. HOT plus NPPV may improve the prognosis of PPFE patients with SRH, although the risk of pneumothorax and oxygen-induced nocturnal hypoventilation must be accurately assessed. In addition, SRH may modify Aspergillus infection in PPFE.

## Data Availability

All data generated or analyzed during this study are included in this published article.

## Abbreviations

AASM: American Academy of Sleep Medicine
ABG: Arterial blood gas
ATS: American Thoracic Society
BA: Bronchial asthma
BMI: Body mass index
CI: Confidence interval
COPD: Chronic obstructive pulmonary disease
CPA: Chronic pulmonary aspergillosis
CXR: Chest X ray
DL_CO_: Diffusing capacity for carbon monoxide
DL_CO_/V_A_: Diffusing capacity for carbon monoxide/alveolar volume
FEV_1_: Forced expiratory volume in one second
FVC: Forced vital capacity
GAP: Gender-age-physiology
HCO_3_^−^: Hydrogen carbonate in awaking arterial blood gas analysis
HOT: Home oxygen therapy
HR: Hazard ratio
HRCT: High-resolution computed tomography
KL-6: Krebs von den lungen-6
ICSD: International Classification of Sleep Disorder
IIP: Idiopathic interstitial pneumonia
ILD: Interstitial lung disease
IPF: Idiopathic pulmonary fibrosis
NPPV: Non-invasive positive pressure ventilation
PaCO_2_: Arterial carbon dioxide partial pressure in awaking
NSIP: Non-specific interstitial pneumonia
PFT: Pulmonary function test
PPFE: Pleuroparenchymal fibroelastosis
PtCO_2_: Transcutaneous carbon dioxide monitoring during sleep
REM: Rapid eye movement
RV: Residual volume
SRH: Sleep related hypoventilation
TLC: Total lung capacity
UIP: Usual interstitial pneumonia
VC: Vital capacity
